# Impact of UVC-sustained recirculating air filtration on airborne bacteria and dust in a pig facility

**DOI:** 10.1371/journal.pone.0225047

**Published:** 2019-11-07

**Authors:** Lisa Eisenlöffel, Tobias Reutter, Matthias Horn, Simon Schlegel, Uwe Truyen, Stephanie Speck

**Affiliations:** 1 Institute of Animal Hygiene and Veterinary Public Health, University of Leipzig, Leipzig, Germany; 2 REVENTA® GmbH, Horstmar, Germany; 3 Institute for Medical Informatics, Statistics and Epidemiology (IMISE), University of Leipzig, Leipzig, Germany; 4 sterilAir AG, Weinfelden, Switzerland; Michigan Technological University, UNITED STATES

## Abstract

High amounts of aerial pollutants like dust and microorganisms can pose serious health hazards to animals and humans. The aim of the current study therefore was, to assess the efficiency of UVC irradiation combined to air filtration in reducing airborne microorganisms at laboratory scale. In a second part, a UVC-combined recirculating air filtration module (UVC module) was implemented in a small animal facility in order to assess its improvement of air quality with regard to airborne bacteria and dust. Tests at laboratory scale were performed using aerosols of *Staphylococcus* (*S*.) *aureus*, *Actinobacillus pleuropneumoniae*, porcine parvovirus (PPV) and porcine reproductive and respiratory syndrome virus. We varied relative humidity (RH) to evaluate its effect on UVC irradiation efficiency. In addition, viability of pathogens inside the filter material was determined over up to six months. UVC-combined air filtration resulted in a more than 99% reduction of viral and bacterial particles. RH had no influence on UVC efficiency. Viability in the filter matter varied depending on the pathogen used and RH with *S*. *aureus* and PPV being most resistant. In our small pig facility consisting of two separated barns, weekly air measurements were conducted over a period of 13 weeks (10 piglets) and 16 weeks (11 piglets), respectively. Airborne bacterial numbers were significantly lower in the barn equipped with the UVC module compared to the reference barn. On average a reduction to 37% of reference values could be achieved for bacteria, whereas the amount of total dust was reduced to a much lesser extent (i.e. to 78% of reference values). Measures taken in front of and behind the UVC module revealed a reduction of 99.4% for airborne bacteria and 95.0% for total dust. To conclude, recirculating air filtration combined to UVC provided efficient reduction of pathogens at laboratory and experimental scale. The implementation of such devices might improve the overall environmental quality in animal facilities.

## Introduction

Pathogens can be transmitted over the airborne route. This has been impressively demonstrated e.g. for porcine reproductive and respiratory syndrome virus (PRRSV), Foot-and-Mouth-Disease virus, *Coxiella burnetii* and *Mycoplasma hyopneumoniae* which can be transported over several kilometers by wind [1–5]. At laboratory scale, a reduction efficiency of 92% to 99.9% was demonstrated depending on the air-filter type and the viruses or bacteria used for testing [6]. Nevertheless, air filtration is still not commonly used in pig production although it minimizes the risk of introducing airborne pathogens by supply air [5–8] and can be used to reduce pathogen burden from indoor air by recirculating air filtration [9–13]. Moreover, as airborne dust can cause serious health problems in animals and humans and acts as a carrier of pathogens, dust control is an important aspect in pig confinement buildings. Indoor air filtration effectively reduced dust levels in pig barns and improved pig performance which resulted in an earlier marketable state of fattening pigs [11]. In another study on a commercial pig farm mean airborne dust concentration was lowest in a barn with recirculating air filtration resulting in enhanced lung health in animals [14]. However, these findings were not affirmed by a significant reduction of airborne bacteria [14]. The authors concluded that combining UVC irradiation to recirculating air filtration might enhance elimination of airborne microorganisms [14]. UVC irradiation (100–280 nm) is highly mutagenic for microorganisms and its efficacy is dependent on the dose (J/m^2^), which is composed of UVC intensity (W/m^2^) and exposure time (s), the wavelength (254 nm), relative humidity (RH) and the susceptibility of microorganisms to UVC irradiation [15–17]. Through the formation of thymidine dimers, UVC functions as a mutagen and leads to damage of the microbial DNA. Several studies demonstrated the virucidal and bactericidal effectiveness of UVC irradiation [18–23]. Increased UVC resistance of certain bacteria was observed with increasing relative humidity (RH) [16–17] which is of importance for usage in pig husbandry conditions. Air disinfection by UVC irradiation is a new technology in pig production. Therefore, the objectives of this study were: 1) To determine the efficiency of UVC irradiation combined to air filtration in an air filter test chamber at laboratory scale using selected pathogens with high relevance in pig production, and 2) to determine the impact of UVC-combined air filtration on the total amount of airborne bacteria and dust in an animal housing at experimental scale.

## Materials and methods

### Pathogens chosen for air filter tests

*Staphylococcus aureus* (strain DSM 799) was chosen because Gram-positive bacteria account for the majority of airborne bacteria inside animal housings. Further, its methicillin-resistant variant (MRSA) can be found in pig barns in high numbers [24]. *Actinobacillus pleuropneumoniae* (APP) causes acute, sub-acute and chronic respiratory infections in pigs. The APP type strain (DSM 13472) was used for our experiments. We further chose PRRSV and Ungulate protoparvovirus I (formerly known as porcine parvovirus, PPV) because of their high economic impact in swine industry [25–27]. Culture conditions and preparation of test suspensions for the bacteria and PRRSV has been previously described in detail [6]. Briefly, bacterial test suspensions in tryptic soy broth (Carl Roth GmbH + Co. KG, Karlsruhe, Germany) were adjusted to 10^8^−10^9^ colony-forming units (cfu)/ml and PRRSV suspensions grown in MARC-145 cells revealed a titer of 10^5.6^–10^6.1^ tissue culture infectious dose (TCID)_50_/ml. PPV (strain NADL2) was grown in SPEV cells (CCLV-RIE 8; Collection of Cell Lines in Veterinary Medicine, Friedrich-Loeffler-Institute, Greifswald-Insel Riems, Germany) at 37°C without CO_2_. The culture medium was composed of equal parts of MEM Hank`s salts (Life Technologies GmbH, Darmstadt, Germany) and MEM Earle`s salts (Life Technologies GmbH) supplemented with non-essential amino acids (Life Technologies GmbH), sodium pyruvate (Life Technologies GmbH), 10% fetal calf serum (FCS; Sigma-Aldrich Chemie GmbH, Schnelldorf, Germany) and L-Glutamine (GlutaMax-l (100x), Life Technologies GmbH). PPV suspensions had a titer of 10^6.8^–10^7.0^ TCID_50_/ml.

### Experiments at laboratory scale

**Fig 1:**
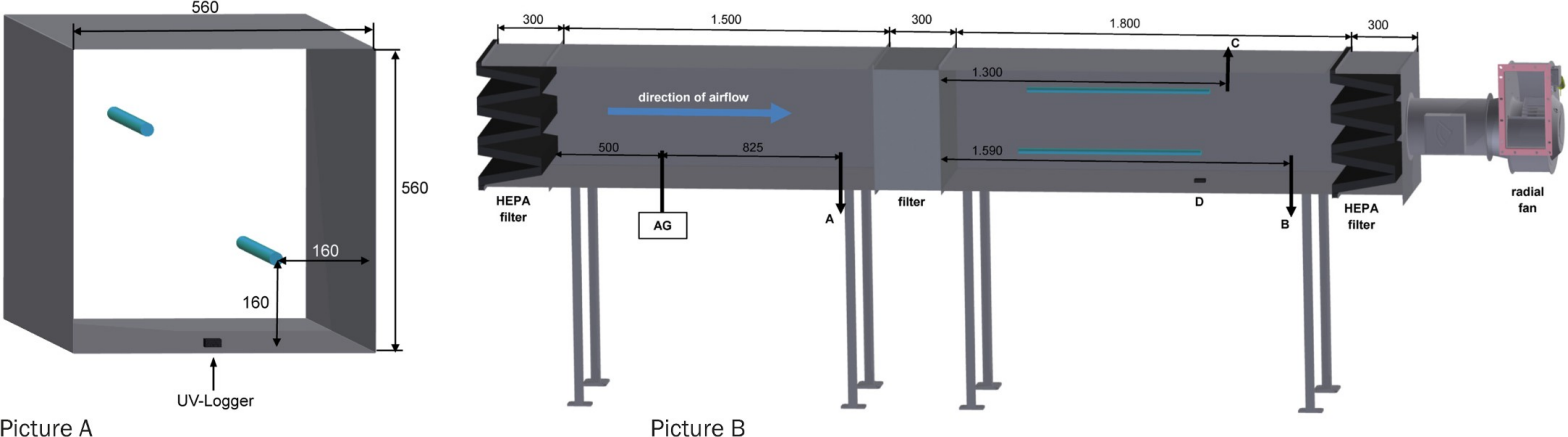
Scheme of the air filter test chamber. (Picture A) Cross section of the test chamber showing the position of both UVC tubes. (Picture B) Longitudinal section of the test chamber. The direction of airflow through the chamber is indicated by a blue arrow. The position of UVC tubes is given by two horizontal blue lines. Dimensions of the test chamber are given in mm. AG: aerosol generator, A: sampling point in front of the air filter, B: sampling point behind the air filter, C: measuring point for relative humidity in the test chamber, D: position of UV-logger.

The air filter test chamber was previously described in detail [6]. Briefly, the chamber had a size of 4,200 mm (length) x 560 mm (width) x 560 mm (height) and was made of galvanized sheet metal with a matt non-reflecting surface. In addition to the original setup the test chamber was equipped with two UVC tubes (2036-4K, 36 W; sterilAir^®^, Weinfelden, Switzerland) of 842 mm in length. They were set horizontally behind the air filter (Fig 1). The commercial air filter used consisted of six filter pockets of polyester (HSB 25A6-3; AFPRO filters GmbH, Bönen, Germany). The filter was classified as ISO Coarse 50% according to DIN EN ISO 16890 [28] or G3-filter according to EN 779:2012 [29], respectively. It had been determined to be <50% efficient at removing particles smaller than 10 μm in diameter. The filter dimension is 592x592x360 mm corresponding to a surface of 2.8 m^2^. The initial pressure loss given by the manufacturer is 30 Pa at 3,400 m^3^/h. The filter was not neutralized in an isopropanol vapor atmosphere [28] as this would impair pathogen viability. Devices for charge neutralization (as aerosolized bacteria may carry electric charges) were not available for our study.

Fifty ml of pathogen suspensions were aerosolized by an Atomizer Aerosol Generator ATM 230 (Topas GmbH, Dresden, Germany), which functions as a collision nebulizer and had been successfully used in a previous study [6]. A particle impaction section removed coarse spray droplets resulting in a particle size distribution of 0.2 μm to 1 μm. HEPA-filtered compressed air of 5 bar was used to produce aerosols supplied into the test chamber. Five independent test runs were performed with each pathogen without and with UVC irradiation, respectively. UVC irradiation (μW/cm^2^) was measured using a UV Microlog MOD_10_ (sglux SolGel Technologies GmbH, Berlin, Germany). The position of the UV-logger is given in Fig 1. Moreover, ultraviolet germicidal irradiation (UVGI) within the filter test chamber was simulated using the software program "sterilAir UVGI" Version 1.0.1.1. (sterilAir^®^, Weinfelden). We repeated tests with *S*. *aureus*, APP, and PRRSV with enhanced RH in the test chamber. This was achieved by enhancing RH in the laboratory using a humidifier. RH (%) inside the air filter test chamber was measured by a Testo 635 (Testo SE & Co. KGaA, Lenzkirch, Germany). Trials with PPV and higher RH were not performed as parvoviruses are known to be very resistant to environmental factors [30]. The test chamber and UVC tubes were turned on for a 20 min warm-up time to achieve a steady-state operating temperature, a constant flow rate and irradiance. Each run was done over a period of 20 minutes. Filters were changed between the pathogens, between runs without and with UVC irradiation, and before tests with enhanced RH. For all tests a volume flow rate of 1,800 m^3^/h was used. The air inlet and outlet of the test chamber was filtered by HEPA filters (class E13) to ensure clean air. Isokinetic air sampling was done and corresponded to a collection volume of 660 l air/h. Air samples were collected by an air sampler pump (Analyt-MTC GmbH, Müllheim, Germany) using water soluble gelatin filters (Sartorius 12602-80-ALK, Sartorius AG, Göttingen, Germany) [6]. The first sampling point was in front of the pocket filter and the second was behind the UVC tubes (Fig 1). Briefly, gelatin filters were dissolved in 5 ml of tryptic soy broth or cell culture medium at 37°C. Determination of bacterial numbers and virus titers was done by the spread-plate method and virus titration, respectively [6,31–32]. For PPV an indirect immunofluorescence was used to determine PPV-infected SPEV cells seven days *post infectionem*. Cells were fixed with ice-cold acetone/methanol (1+1, v/v) and then rehydrated using PBS (pH 7.4). After blocking the cells with 3% FCS (Sigma-Aldrich Chemie GmbH) a PPV-positive swine serum (1:100) was applied and incubated at 37°C overnight. The serum was washed off with PBS and a fluorescein-conjugated second antibody (FITC-conjugated AffiniPure Goat Anti-Swine IgG, Jackson ImmunoResearch Laboratories, Inc., West Grove, Pennsylvania, USA) was added. After 8 hours of incubation at 37°C the titer plates were analyzed by a DMIL fluorescence microscope (Leica, Wetzlar, Germany) and the TCID_50_/ml was calculated. The reduction efficiency of pathogens was calculated according to the following equation:

$$Reduction\;(\%)=\frac{pathogen\;number\;behind\;the\;filter\;and\;UVC\;tubes}{pathogen\;number\;in\;front\;of\;the\;filter}*100\%$$

To investigate pathogen survival in the filter matter, each filter was placed into a separate plastic bag and stored at room temperature subsequently after the experiment. Five samples of 1 cm^2^ each per filter were cut out after certain time intervals (30 min, 60 min, 120 min, 240 min, 24 h, 48 h, 7 d, 4 weeks, 2 months and 6 months), pooled and incubated for 10 min at room temperature in the respective culture medium. Virus titration and counting of bacteria was done as described before. All samples were additionally stored at -80°C. Prior to the experiments the filter material had been tested on all used cell lines and bacteria and revealed no toxic effects.

### Description of the animal housing

**Fig 2:**
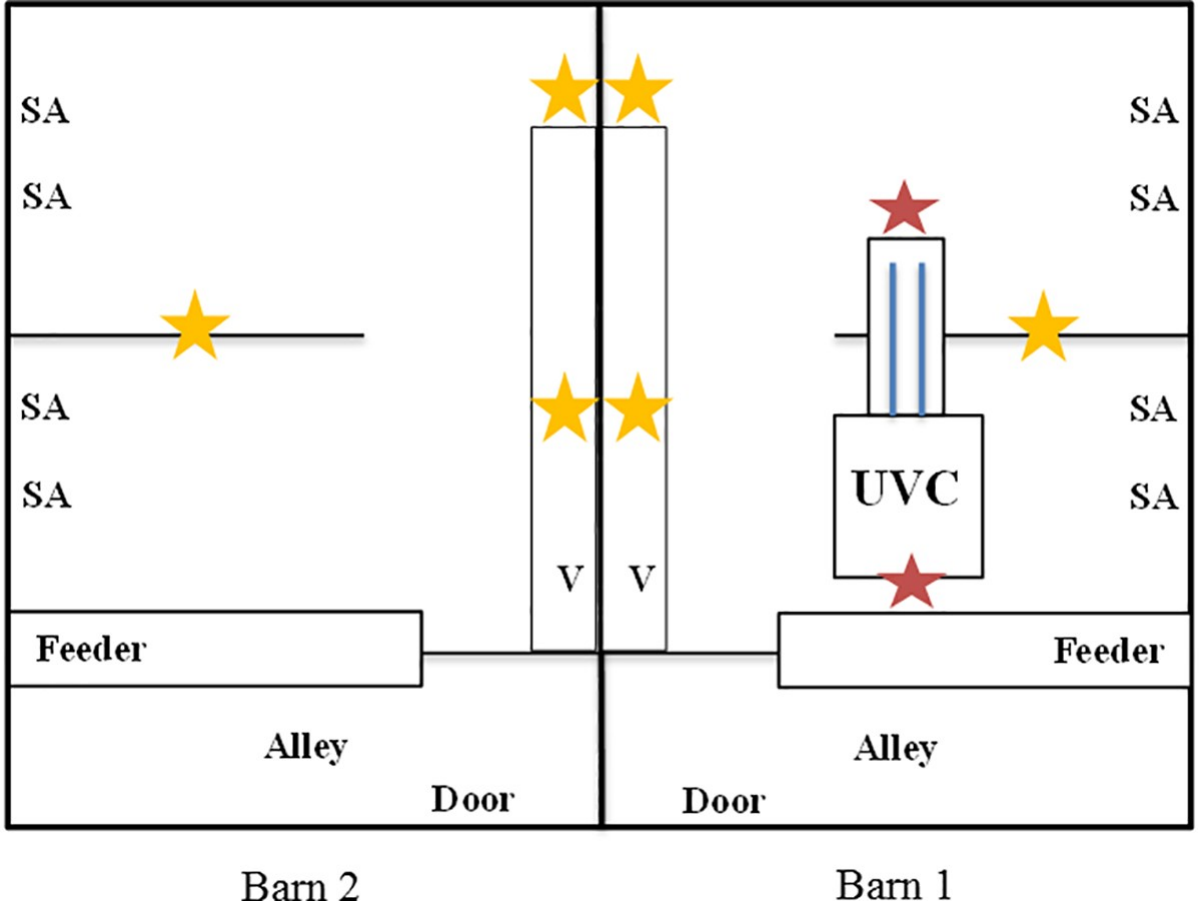
Structure of the barns and sampling points. Stars in yellow indicate the sampling locations for airborne dust and bacteria. Measures taken in front of and behind the UVC module are given as light red stars. SA–inlet valves for supply air; UVC–position of the UVC module; V–position of exhaust air vents.

**Fig 3:**
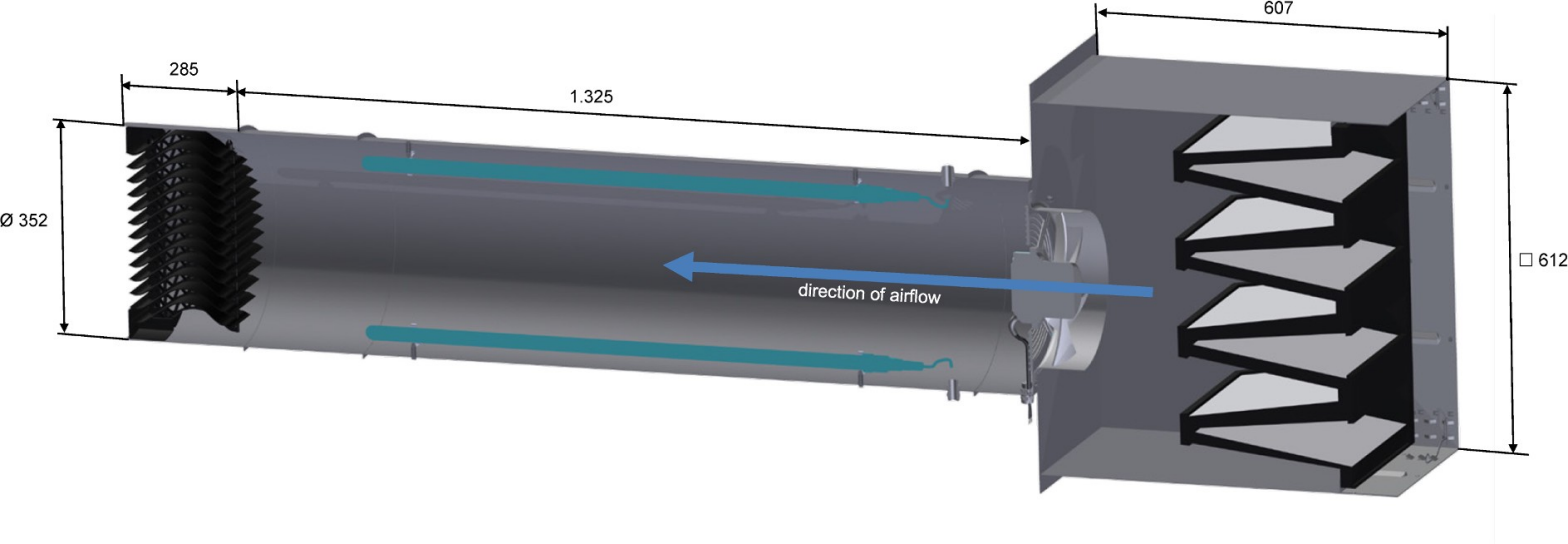
Longitudinal section of the UVC module. The module was composed of a pocket air filter (right rim of the picture), an axial fan (between filter and UVC tubes), and four UVC tubes (indicated as blue lines). A protective grid located downstream (left rim) ensured protection against UVC irradiation. The direction of airflow is given as blue arrow. UVC module dimensions are given in mm.

The animal housing belongs to the Faculty of Veterinary Medicine at Leipzig University and consisted of two similar barns (barn 1: 7.23 m length x 3.32 m width x 2.92 m height; barn 2: 7.23 m length x 3.30 m width x 2.92 m height) arranged in parallel. Each barn was entered through a separate hygiene sluice were rubber boots were changed. The barns were equipped with separated air conditioning systems. Filtered (compact filter, class F7, DELBAG FläktGroup Deutschland GmbH, Herne, Germany) fresh air was delivered into each barn via four inlet valves at the ceiling (Fig 2). Windows remained closed for the entire study. The ventilation flow rate (equal pressure ventilation) was 1,200 m^3^/h resulting in approximately 17 times air change per hour. Used air was exhausted from each barn via four vents which were located at animal level behind a partition wall on one longitudinal site. Husbandry conditions were according to the German regulation for the protection of farmed animals [33]. Animals were housed with permission granted by the Landesdirektion Sachsen (reference number DD24-5131/244/11). Barn 1 was additionally equipped with a UVC-recirculating air filtration module (UVC module) (Fig 2). The UVC module consisted of the coarse dust filter tested at laboratory scale and two UVC tubes (2036-4K, sterilAir^®^) located behind the filter (Fig 3). Continuous air flow was guaranteed by an axial fan and was adjustable by a potentiometer. The ventilation flow rate of the UVC module in barn 1 additionally achieved a six times air change per hour. The air filter of the UVC module was changed between the first and the second trial of the animal study.

### Animals and husbandry conditions

Pigs were obtained from the Teaching and Research Farm of the University of Leipzig (registration number according to the German Livestock Movement Order “Viehverkehrsverordnung” DE143792903000). Animals were housed with permission granted by the Landesdirektion Sachsen (reference number DD24-5131/244/11). Husbandry conditions were according to the German regulation for the protection of farmed animals. The study was approved by the Animal Welfare Officer of the Veterinary Faculty at Leipzig University. According to the German Animal Protection Law, our animal study was not defined as an animal experiment and, therefore, an approval by the Committee on the Ethics of Animal Experiments was not necessary. At the end of each trial animals were transported to slaughter according to the German legislation regarding animal protection during transport (Tierschutztransportverordnung).

Piglets were housed on fully slatted floors without litter. Two trials over a period of 13 and 16 weeks respectively were performed. In the first trial 10 animals were kept per barn. Drinking water was provided by nipple drinkers and animals were fed a dry industry-standard diet *ad libitum* using manually filled feeders. Eleven piglets per barn were included in the second trial and were fed the same diet but only twice a day. Slurry trays underneath the slatted floors were discontinuously discharged in the middle and at the end of each trial. Each barn was cleaned and disinfected before restocking. Personnel had to change clothes before entering the animal housing. Animal health and behavior was checked daily by observation with particular attention to respiratory symptoms. Lung health was examined post-mortem by slaughterhouse personnel and results reported.

### Measurement of indoor air quality

**Fig 4:**
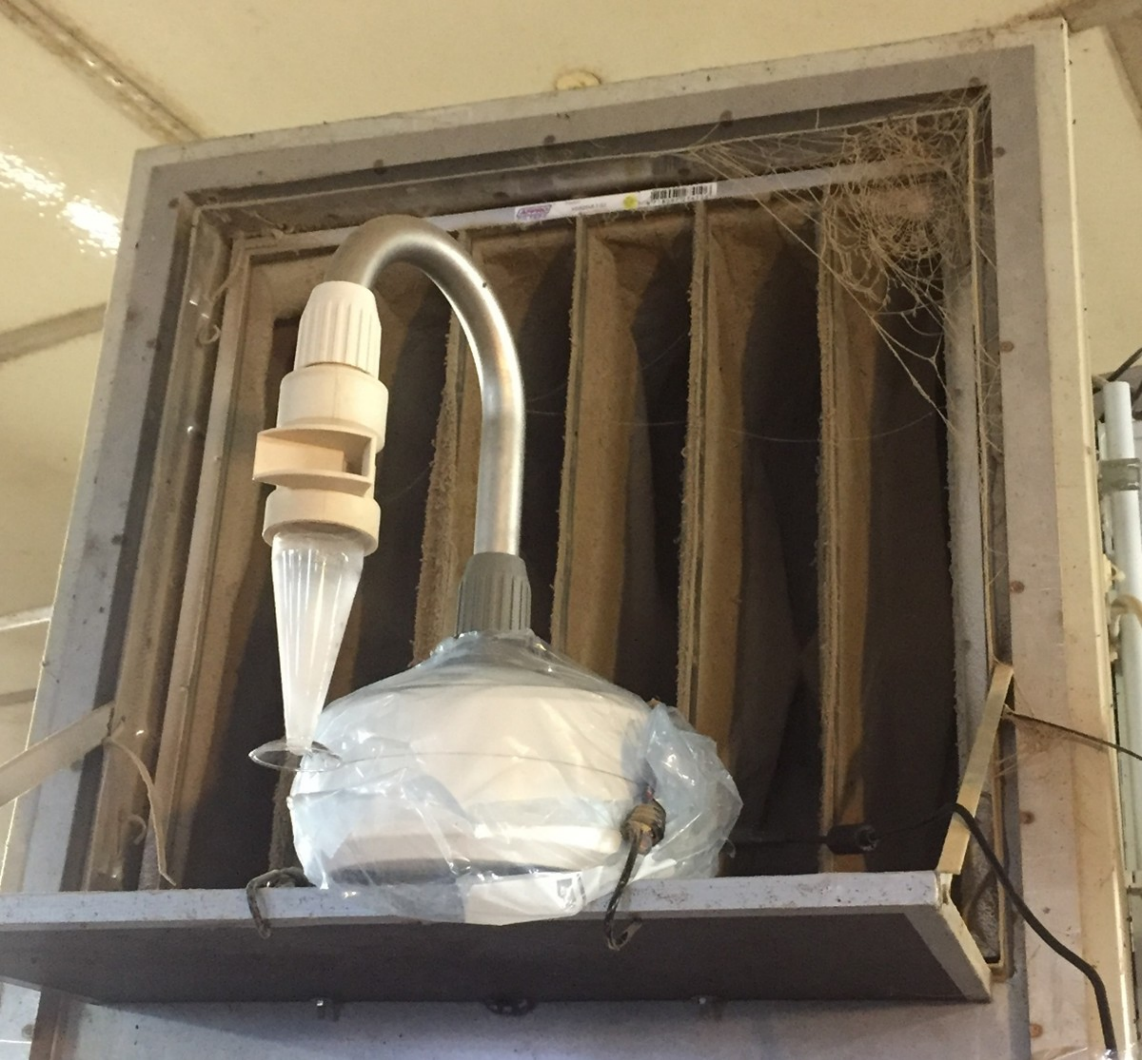
Position of the Coriolis^®^μ Air Sampler in front of the UVC module. The picture shows the Coriolis^®^μ Air Sampler operating on a mounted board in front of the pocket filter for measurements in close proximity to the UVC module.

Measurements were conducted weekly between 9:00 a.m. and 12:00 p.m.. Dust values were calculated from data collected over 10 min sampling time using the DustTrak^TM^ DRX Aerosol Monitor 8533 (TSI GMbH, Aachen, Germany), which is able to distinguish particle size fractions of ≤1 μm, ≤2.5 μm, ≤4 μm, ≤10 μm and a total size fraction. Airborne bacteria were sampled by a Coriolis®μ Air sampler (Bertin Technologies, Montigny le Bretonneux, France) and samples were processed as described previously [14]. In addition, ammonia (Model CMS; Dräger Safety AG & Co. KGaA, Lübeck, Germany), CO_2_ (Testo 535), temperature and RH (EBI 20-TH-1 data logger; Xylem Analytics Germany Sales GmbH & Co. KG ebro, Ingolstadt, Germany) were recorded. Dust and airborne bacteria were first sampled only at one sampling location per barn. Instruments were placed at an approximately height of 1.0 m. As of the third week of trial 1, dust was sampled at two locations. In the second trial, all samplings were carried out at three sampling locations (Fig 2). To avoid false high values due to an increased pig activity, feeders were filled at least one hour before measurements were taken during the first trial. In the second trial feeding was done afterwards. In addition, the effect of UVC-combined air filtration was assessed by comparing values simultaneously measured in front of (air inlet) and behind (air outlet) the UVC module (Fig 4) at a height of 1.93 m. Personnel left the barn during measurements.

### Statistical analyses

All statistical analyses were performed using Microsoft® Excel® 2019. Barn 2 without any additional air filtration was set as the reference. Measurements of airborne bacteria and dust amounts were assumed to be independent of all previous measurements. Only those values obtained with the UVC module in operation were included in the statistical analyses, i.e. n = 12 timepoints for the first and n = 16 for the second trial. For each trial, the ratio of airborne bacterial counts in barn 1 (UVC module) compared to barn 2 (reference) was calculated (mean bacterial numbers in barn 1 divided by mean bacterial numbers in barn 2). Ratios smaller than one correspond to a relative reduction of bacterial counts. Changes of dust amounts with respect to the reference were calculated in a similar manner. Ratios were log-transformed to guarantee approximately normally distributed values. For each trial, the null hypotheses of unchanged mean bacterial or dust counts (i.e., mean ratios equal one) were tested using one sample t-tests on a significance level of 5%. In addition, 95% confidence intervals (CI) were calculated for the estimated sample means.

## Results

### Retention efficiency determined in an air filter test chamber

The results of the different test runs are summarized in Table 1. Irrespective of the pathogen used a high loss of infectious particles was noticed when comparing pathogen numbers filled into the atomizer and pathogen amount measured in front of the filter (Table 1). Overall the results show that the G3-filter accounted for a low reduction of infectious particles with high variation within the five runs. Using UVC irradiation alone or in combination to the air filter resulted in a more than 99% reduction for bacteria and viruses. Only marginal differences were seen between the five replicates for each pathogen. The most efficient reduction was achieved for APP (i.e. 100%). In the first experiments RH was not modified and varied markedly. In the second test series, experiments with *S*. *aureus*, APP and PRRSV were repeated with RH adjusted to a mean of 66% (Table 2). Higher RH resulted in higher reduction rates for all pathogens in air filter tests without UVC irradiation. The effectiveness of the latter was not affected by higher RH. Simulation of UVGI at a volume flow rate of 1,800 m^3^/h, 20°C within the test chamber revealed an average intensity of 1,612 μW/cm^2^ at 20% RH (S1 File) and 66% RH (S2 File), respectively. This is similar to the results measured by the UV-logger (Tables 1 and 2).

**Table 1:** Retention determined in an air filter test chamber with and without UVC irradiation.

Pathogen	Test setup	UVC intensity (μW/cm^2^)	RH (%)^a^	Pathogen amount			Reduction (%) ± SD^b^
				in culture suspension filled into the atomizer	in front of the filter	behind the filter	
***S*. *aureus***	**filter**	0	15.3	7.34 x 10^8^ cfu/ml	8.31 x 10^5^ cfu/ml	2.64 x 10^5^ cfu/ml	67.71 ± 7.41
	**filter, UVC**	1,100	3.2^c^	6.73 x 10^8^ cfu/ml	3.43 x 10^5^ cfu/ml	1.13 x 10^3^ cfu/ml	99.74 ± 0.57
	**UVC**	1,190	12.7	6.42 x 10^8^ cfu/ml	5.07 x 10^5^ cfu/ml	4.60 x 10^1^ cfu/ml	99.99 ± 0.01
**APP**	**filter**	0	15.3	8.73 x 10^8^ cfu/ml	1.49 x 10^3^ cfu/ml	7.63 x 10^2^ cfu/ml	49.43 ± 23.77
	**filter, UVC**	1,220	17.4	9.40 x 10^8^ cfu/ml	9.15 x 10^2^ cfu/ml	0 cfu/ml	100.0 ± 0.0
	**UVC**	1,180	15.4	8.10 x 10^8^ cfu/ml	5.53 x 10^3^ cfu/ml	0 cfu/ml	100.0 ± 0.0
**PRRSV**	**filter**	0	nd ^c^	10^5.8^ TCID_50_/ml	10^3.5^ TCID_50_/ml	10^2.7^ TCID_50_/ml	74.19 ± 16.70
	**filter, UVC**	1,160	33.8	10^5.7^ TCID_50_/ml	10^3.8^ TCID_50_/ml	10^1.0^ TCID_50_/ml	99.65 ± 0.41
	**UVC**	1,170	19.0	10^6.1^ TCID_50_/ml	10^3.7^ TCID_50_/ml	10^1.3^ TCID_50_/ml	99.29 ± 0.82
**PPV**	**filter**	0	37.9	10^7.0^ TCID_50_/ml	10^4.8^ TCID_50_/ml	10^4.1^ TCID_50_/ml	78.71 ± 17.70
	**filter, UVC**	1,180	43.2	10^6.8^ TCID_50_/ml	10^4.8^ TCID_50_/ml	10^1.7^ TCID_50_/ml	99.82 ± 0.12
	**UVC**	1,170	32.8	10^6.8^ TCID_50_/ml	10^4.6^ TCID_50_/ml	10^1.5^ TCID_50_/ml	99.87 ± 0.10

nd–not measured

SD–standard deviation

^a^RH in these experiments was not modified and was documented once per run in the middle of the sampling time (i.e. after 10 min).

^b^Results represent the mean reduction (%) of five independent runs ± SD.

^c^ device defective

**Table 2:** Influence of relative humidity on the reduction efficiency of UVC-combined air filtration at laboratory scale.

Pathogen	Test setup	UVC intensity (μW/cm^2^)	RH (%)^a^	Pathogen amount			Reduction (%) ± SD^b^
				in culture suspension filled into the atomizer	in front of the filter	behind the filter	
***S*. *aureus***	**filter**	0	67.1	7.50 x 10^8^ cfu/ml	8.10 x 10^5^ cfu/ml	1.50 x 10^5^ cfu/ml	80.88 ± 4.23
	**filter, UVC**	1,260	66.3	5.50 x 10^8^ cfu/ml	5.58 x 10^5^ cfu/ml	3.75 x 10^0^ cfu/ml	99.99 ± 0.00
**APP**	**filter**	0	65.5	7.60 x 10^8^ cfu/ml	3.10 x 10^4^ cfu/ml	6.60 x 10^3^ cfu/ml	79.48 ± 3.51
	**filter, UVC**	1,220	65.9	7.60 x 10^8^ cfu/ml	2.20 x 10^4^ cfu/ml	0 cfu/ml	100.0 ± 0.0
**PRRSV**	**filter**	0	65.6	10^6.1^ TCID_50_/ml	10^3.8^ TCID_50_/ml	10^3.0^ TCID_50_/ml	75.99 ± 10.76
	**filter, UVC**	1,180	65.7	10^5.5^ TCID_50_/ml	10^3.1^ TCID_50_/ml	10^1.1^ TCID_50_/ml	98.63 ± 1.47

SD–standard deviation

^a^ RH was documented once per run in the middle of the sampling time (i.e. after 10 min).

^b^Results represent the mean reduction (%) of five independent runs ± SD.

Viability of pathogens in the filter material from the first experiments was best for PPV which stayed infectious at least for four weeks but was no longer cultivable after two months. *S*. *aureus* lost its viability after one week while the filter sustained viability of PRRSV at least for 24 hours. In contrast, APP was cultivable only until 30 min after the end of the run. Increasing RH extended the viability of APP (4 h) and *S*. *aureus* (2 months) in the filter material while PRRSV seemed less viable (Table 3).

**Table 3:** Survival of bacteria and viruses in the filter matter determined after selected points in time.

Pathogen	Test setup	Pathogen viability after selected points in time									
		0.5 h	1 h	2 h	4 h	24 h	48 h	1 week	4 weeks	2 months	6 months
***S*. *aureus***	**filter**	+	+	+	+	+	+	+	-	nd	-
	**filter, UVC**	+	+	+	+	+	+	+	-	nd	-
	**filter, UVC, RHadj**	+	+	+	+	+	+	+	+	+	nd
**APP**	**filter**	+	-	-	-	-	-	-	-	nd	-
	**filter, UVC**	-	-	-	-	-	-	-	-	nd	-
	**filter, UVC, RHadj**	+	+	+	+	-	-	nd	nd	nd	nd
**PRRSV**	**filter**	+	-	+	+	+	-	-	-	nd	nd
	**filter, UVC**	+	+	+	+	+	-	-	-	nd	nd
	**filter, UVC, RHadj**	+	+	+	+	-	-	nd	nd	nd	nd
**PPV**	**filter**	+	+	+	+	+	+	+	+	-	nd
	**filter, UVC**	+	+	+	+	+	+	+	+	-	nd
	**filter, UVC, RHadj**	nd	nd	nd	nd	nd	nd	nd	nd	nd	nd

RHadj–adjusted relative humidity (66%±0.6%)

nd–not determined

+ indicates bacterial/viral growth

- indicates no bacterial/viral growth

### Bacteria and total dust concentration in air samples

Results are displayed in Table 4. Measures performed before restocking (i.e. after cleaning and disinfection) revealed total bacterial counts of ≤110 cfu/m^3^ and a total dust amount of <0.02 mg/m^3^.

**Table 4:** Amount of total dust and bacteria obtained from air samplings.

Trial	Season	Week	No. of animals	Ventilation flow rate, UVC module (m^3^/h)	Total dust (mg/m^3^ ± SD)		Total airborne bacteria (cfu/m^3^ ± SD)	
					Barn 1 with UVC module	Barn 2 (Reference)	Barn 1 with UVC module	Barn 2 (Reference)
1	Spring	0	0	na	0.014^a^	0.016 ^a^	0^b^	0^b^
		1	10	na	0.341 ^a^	0.586 ^a^	56,000^b^	290,000^b^
		2*	10	416	0.599 ^a^	0.477 ^a^	93,000^b^	46,000^b^
		3*	10	409	0.288 ± 0.206	0.581 ± 0.105	38,000^b^	140,000^b^
	Summer	4*	10	385	0.485 ± 0.408	0.650 ± 0.126	48,000^b^	110,000^b^
		5*	10	341	0.380 ± 0.207	0.458 ± 0.353	92,000^b^	320,000^b^
		6	10	423	0.365 ± 0.003	0.131 ± 0.016	140,000^b^	420,000^b^
		7	10	359	0.172 ± 0.115	0.513 ± 0.401	430,000^b^	750,000^b^
		8	10	315	0.302 ± 0.130	0.367 ± 0.255	240,000^b^	620,000^b^
		9	10	357	0.282 ± 0.146	0.228 ± 0.243	1,300,000^b^	1,100,000^b^
		10	10	322	0.607 ± 0.301	0.230 ± 0.018	780,000^b^	540,000^b^
		11	10	366	0.610 ± 0.323	1.340 ± 0.156	1,000,000^b^	310,000^b^
		12	10	300	0.710 ± 0.595	0.879 ± 0.482	510,000 ± 42,400	570,000 ± 127,000
		13	9	352	0.363 ± 0.263	0.738 ± 0.291	345,000 ± 63,600	820,000 ± 396,000
2	Summer	0	0	0	0.010 ± 0.005	0.004 ± 0.001	84 ± 23	40 ± 13
	Autumn	1	11	405	0.468 ± 0.112	0.597 ± 0.144	287,000 ± 49,300	397,000 ± 130,000
		2	11	405	0.200 ± 0.103	0.545 ± 0.117	81,000 ± 26,200	297,000 ± 66,600
		3	11	357	0.295 ± 0.079	0.603 ± 0.186	73,700 ± 15,500	267,000 ± 102,000
		4	11	387	0.667 ± 0.243	0.530 ± 0.055	94,300 ± 41,800	200,000 ± 60,800
		5	11	402	0.548 ± 0.214	0.403 ± 0.113	91,700 ± 18,500	433,000 ± 222,000
		6	11	392	0.643 ± 0.064	0.865 ± 0.250	190,000 ± 62,400	1,060,000 ± 661,000
		7	11	394	0.672 ± 0.086	0.890 ± 0.249	187,000 ± 95,000	530,000 ± 303,000
		8	11	393	0.848 ± 0.198	0.845 ± 0.207	141,000 ± 58,500	433,000 ± 115,000
		9	11	402	0.999 ± 0.374	1.360 ± 0.262	190,000 ± 60,800	527,000 ± 246,000
		10	11	420	0.572 ± 0.369	1.757 ± 0.567	53,700 ± 37,200	1,000,000 ± 195,000
		11	11	392	0.817 ± 0.250	1.257 ± 0.176	217,000 ± 70,900	1,070,000 ± 405,000
		12	11	422	1.281 ± 0.401	1.042 ± 0.548	260,000 ± 161,000	557,000 ± 169,000
		13	11	383	1.327 ± 0.309	0.855 ± 0.699	250,000 ± 115,000	130,000 ± 98,900
	Winter	14	11	427	0.831 ± 0.485	1.437 ± 0.367	80,000 ± 36,100	217,000 ± 37,900
		15	11	441	1.440 ± 0.427	1.867 ± 0.540	460,000 ± 105,000	520,000 ± 504,000
		16	11	456	1.320 ± 0.708	1.270 ± 0.480	410,000 ± 26,900	433,000 ± 90,700

Measurements were taken weekly. The ventilation flow rate at the UVC module was read at the beginning of each sampling day. Total dust values (mean ± SD) were calculated from data collected by the DustTrak^™^ DRX Aerosol Monitor over 10 min. Total dust comprises particles ≤1 μm, ≤2.5 μm, respirable particles, and ≤10 μm. The amount of total bacteria represents the mean ± SD of measurements done at three sampling locations (indicated as yellow stars in Fig 2). UVC intensity was between 1,100 and 1,280 μW/cm^2^. Seasons were defined according to the astronomical calendar: spring (21^st^ March to 20^th^ June), summer (21^st^ June to 22^nd^ September), autumn (23^rd^ September to 21^st^ December), winter (22^nd^ December to 20^th^ March).

na–Not applicable as the module had been installed the second week of the first trial.

*Climate control unit of the general ventilation system in barn 1 was defective.

^a^ During the first two weeks of trial 1, dust was measured only at one sampling location.

^b^During the first trial, bacterial counts were measured only at one sampling location.

In the first trial, total airborne bacterial numbers exceeded 10^6^ cfu/m^3^ in both barns. Total dust was always below 1 mg/m^3^ in barn 1 and only once higher in barn 2. Overall, bacteria and dust in barn 1 could be reduced to an average of 68.4% (95% CI [40.6%,115%]) and 86.5% (95% CI [56.7%,132%]), respectively, compared to the reference barn 2. The observed reductions were not statistically significant (p = 0.14 for bacteria and p = 0.46 for dust).

In the second trial, bacterial numbers exceeded 10^6^ cfu/m^3^ only in barn 2. With the exception of the results obtained at week 13, the bacterial numbers in air samples were always lower in barn 1 compared to barn 2. The amount of airborne dust was higher compared to the first trial and increased to >2 mg/m^3^ in barn 2 but was always less than 2 mg/m^3^ in barn 1. Bacteria and dust in barn 1 could be reduced to an average of 37.0% (95% CI [23.9%,57.2%]) and 78.0% (95% CI [61.3%,99.1%]), respectively, compared to the reference barn 2. These reductions were statistically significant (p = 0.0002 for bacteria and p = 0.04 for dust).

Measures taken in front of and behind the UVC module simultaneously (Table 5) revealed a reduction of 99.4% for airborne bacteria which is similar to the results at laboratory scale and 95.0% for total dust. UVC intensity determined inside the module adjacent to the air outlet ranged from 1,100 to 1,280 μW/cm^2^. Air velocity at the outlet was 1.10 m/s and 1.20 m/s on average in the first and second trial, respectively. Overall, concentrations of airborne bacteria determined at the height of the UVC module were lower compared to those measured at the sampling points within the barn.

**Table 5:** Amount of total dust and bacteria measured at the inlet and outlet of the UVC module in barn 1.

Trial	Season	Week	No. of animals	Ventilation flow rate, UVC module (m^3^/h)	Total dust (mg/m^3^ ± SD)		Total airborne bacteria (cfu/m^3^ ± SD)	
					in front of UVC module	behind UVC module	in front of UVC module	behind UVC module
1	Spring	0	0	na	na	na	na	na
		1	10	na	na	na	na	na
		2	10	416	0.222	0.060	78,000	10,000
		3	10	409	0.225	0.035	30,000	1,300
	Summer	4	10	385	0.361	0.026	42,000	530
		5	10	341	0.155	0.013	80,000	380
		6	10	423	0.436	0.013	130,000	560
		7	10	359	0.100	0.003	740,000	600
		8	10	315	0.398	0.004	270,000	580
		9	10	357	0.439	0.003	480,000	950
		10	10	322	0.378	0.003	280,000	950
		11	10	366	0.119	0.001	710,000	600
		12	10	300	0.266	0.001	110,000	490
		13	9	352	0.354	0.009	190,000	1,000
2	Summer	0	0	0	module set off	module set off	module set off	module set off
	Autumn	1	11	405	0.257	0.071	400,000	7,900
		2	11	405	0.217	0.047	120,000	540
		3	11	357	0.629	0.029	140,000	350
		4	11	387	0.502	0.035	170,000	1,000
		5	11	402	0.308	0.014	150,000	790
		6	11	392	0.215	0.010	110,000	1,400
		7	11	394	0.435	0.031	130,000	800
		8	11	393	0.449	0.014	150,000	1,200
		9	11	402	0.318	0.008	250,000	4,200
		10	11	420	0.533	0.010	71,000	1,000
		11	11	392	0.296	0.007	180,000	530
		12	11	422	0.637	0.014	580,000	700
		13	11	383	0.696	0.005	730,000	2,400
	Winter	14	11	427	0.387	0.004	110,000	610
		15	11	441	0.759	0.015	210,000	7,700
		16	11	456	0.620	0.004	260,000	600

Measurements were taken weekly. The ventilation flow rate at the UVC module was read at the beginning of each sampling day. Total dust values (mean ± SD) were calculated from data collected by the DustTrak^™^ DRX Aerosol Monitor over 10 min. Total dust comprises particles ≤1 μm, ≤2.5 μm, respirable particles, and ≤10 μm. UVC intensity was between 1,100 and 1,280 μW/cm^2^. Seasons were defined according to the astronomical calendar: spring (21^st^ March to 20^th^ June), summer (21^st^ June to 22^nd^ September), autumn (23^rd^ September to 21^st^ December), winter (22^nd^ December to 20^th^ March).

na–Not applicable as the module had been installed in the second week of the first trial.

### Indoor temperature, relative humidity, CO_2_, and NH_3_

During the first trial, indoor temperature varied from 20.0°C to 32.8°C (mean of 24.3°C, barn 1) and from 16.8°C to 32.8°C (mean 23.3°C, barn 2), respectively. Relative humidity indoors ranged from 24.2% to 83.6% (mean 51.9%) in barn 1 and from 25.7% to 92.9% (mean 58.6%) in barn 2. The mean concentration of CO_2_ was 1,029 ppm (738–1,325 ppm, barn 1) and 1,013 ppm (808–1,146 ppm, barn 2), respectively. Ammonia ranged from <2.0–6.2 ppm in barn 1 and <2.0–15.4 ppm in barn 2.

In the second trial, indoor temperature of barn 1 varied from 15.7°C to 28.6°C (mean 22.5°C) and was similar for barn 2 (17.5°C to 28.6°, mean 22.9°C). Relative humidity indoors ranged from 24.3% to 93.6% (mean 42.3%, barn 1) and from 25.4% to 85.9% (mean 43.1%, barn 2), respectively. The mean concentration of CO_2_ was 1,144 ppm (792–1,718 ppm, barn 1) and 1,100 ppm (749–1,449 ppm, barn 2), respectively. Ammonia ranged from <2.0–9.6 ppm in barn 1 and <2.0–5.8 ppm in barn 2.

### Animal health

Respiratory symptoms were not noticed during both trials of the study. In the last week of the first trial one animal had to be euthanized because of a non-healing hoof wound, which led to lameness, pain and a separation from the group. At daily check-ups we observed that animals kept in barn 1 (with UVC module) overall were more active compared to the animals in barn 2. Lung health determined after the first trial at slaughter revealed pneumonia in one pig from barn 2 (reference barn) and no signs of pulmonary disease in barn 1 (with UVC module). Lung health was not impaired in any pig from the second trial.

## Discussion

Air filters are highly efficient in reducing pathogens at laboratory scale [6]. According to the manufacturer, the efficiency of the coarse dust filter used in this study was less than 50% at removing particles of <10 μm in diameter. Nevertheless, this filter reduced viruses by approximately 74% (PRRSV) and 79% (PPV) from air in our experimental setting. Tests with bacteria resulted in a reduction of 50% (APP) and 68% (*S*. *aureus*), respectively. These results suggest that coarse dust filters may not be suitable to minimize the risk of pathogen introduction via supply air. However, inside animal housings pathogens are mostly associated with dust particles which are easier to trap [34]. Moreover, dust bound to the filter matter will increase the filter retention efficiency over time to a certain degree. Higher numbers of APP passed the filter although APP is larger in size than viruses and *S*. *aureus*. This phenomenon has been described before [6] and yet remains unexplained. Using UVC irradiation in addition to the air filter resulted in a >99% to 100% reduction of viruses and bacteria. This is accordant to others although UVC intensity varied distinctly between our experiments and these studies [17,23,35]. This may be attributed to the single UVC measurement device in our study which was placed at least 160 mm underneath the UVC tubes and measured irradiance at the bottom of the test chamber. However, UVGI simulation revealed an average UVC intensity similar to the results measured by the UV-logger. The spatial distribution of UVC irradiation within the test chamber [17,36] and the effective radiation exposure on airborne pathogens could not be assessed using this device. Moreover, we were not able to determine the total irradiation exposure that a particle would receive during its passage through the test chamber, which must be seen as a major weakness of our study. In addition, the volume flow rate in our experiments was higher compared to studies described elsewhere, hence our results are contradictory to others which assumed that a higher ventilation rate leads to a shorter residence time which could decrease UVC effectiveness [17,23,36]. Our experiments demonstrated that UVC irradiation solely was as effective as UVC combined to air filtration in our setting (i.e. two tubes of 842 mm in length, air filter test chamber with a dimension of 4.2 m length x 0.56 m height x 0.56 m width, volume flow rate 1,800 m^3^/h). Nevertheless, an upstream coarse dust filter seems favorable as it diminishes dust deposition on the UVC tubes which ensures adequate irradiation emission. Differences in susceptibility to UVC irradiation and reduction efficiency between grampositive (*S*. *aureus*) and gramnegative (APP) bacteria, as described elsewhere [17–18,37–40], were not observed. Survival of the pathogens in the filter matter was similar to our previous study [6] except for APP. APP is known to be susceptible to desiccation [41] and viability became undetectable within more than 30 min after the experiment. PPV remained infectious in the filter matter for at least four weeks and it has been described to persist for several months under favorable conditions outside the host [42].

It has been described that the number of microorganisms killed by UVC irradiation decreased when relative humidity increased [16–17,20,22,37,40,43]. This was not supported by our results which rather confirm the findings of Rentschler and Nagy (1940) [44] and Walker et al. (2007) [45] who described no effect of relative humidity on UVC impact. Interestingly, the higher RH resulted in an increased viability in the filter matter of *S*. *aureus* (at least two months) and APP (at least 4 h). After each experiment, filters were subsequently stored in plastic bags that prevented evaporation of the moist inside the filter matter which most likely supported survivability.

Concern has been raised that UVC-inactivation rates determined by culture might be influenced by the type of air sampler [17]. Our experiments used an air sampler pump, which implies less stress for bacteria compared to impingement, and water-soluble gelatin membrane filters [6]. The latter offer a high retention rate and protect sampled microorganisms from drying. Bacteria may also experience stress during aerosolization by collision nebulizers such as the ATM 230, possibly resulting in false high reduction efficiencies [6,46]. We calculated reduction rates from aerosolized pathogens sampled in front of and behind the air filter hence, pathogens in both samples were likewise impaired by aerosolization and retention. The effect of both might therefore be negligible. Moreover, aerosolized bacteria carry electric charges which play an important role when particles are collected on filters [47]. Devices for charge neutralization were not available for our study. Moreover, the filter could not be neutralized in an isopropanol vapor atmosphere to eliminate electrostatic charge [6].

High amounts of aerial pollutants including dust and microorganisms can pose serious health hazards to animals and humans [48–50]. Microbial pollutants and dust in confined pig housings mainly originate from animals (skin, feces), bedding, and feed. Reducing dust indoors will concomitantly reduce microorganisms associated to dust [48]. However, although it has been described that high dust levels in pig housings reduce pig performance, measures for dust reduction are not commonly implemented [9,34]. As a second part of the present study, the efficiency of UVC irradiation combined to recirculating air filtration in a small pig facility was evaluated. The number of airborne bacteria determined was within the range of other studies [12,14,48,50]. Under conditions of our study, a mean removal efficiency of 31.6% to 63% for airborne bacteria in barn 1 compared to the reference barn was achieved which supports findings of others [51,52]. However, this reduction was less than the results obtained for *S*. *aureus* and APP at laboratory scale. This can be explained by the varying UVC-sensitivity of different airborne bacterial species as has been described e.g. for spore-forming *Bacillus* species which can be frequently found in environmental samples [53]. We used similar air filtration modules without UVC in a previous study without a remarkable reduction of airborne bacteria [14], hence, the reduction efficiency achieved here cannot only be attributed to the additional six times air change of the air filtration module. Significantly lower bacterial numbers in barn 1 compared to the reference barn could only be achieved in the second trial. This might in part be explained by the fact that the piglets in trial 1 were older at restocking compared to piglets in trial 2. Therefore, the fattening period was shorter (i.e. 13 weeks) and less data were collected compared to the second trial. The smaller number of measurements (n = 12) as compared to the second trial (n = 16) resulted in a higher inaccuracy of reduction estimates. Moreover, a moderate fly infestation had to be eradicated during the first trial. The movements of the flies may have raised settled dust into suspension thereby also increasing the number of airborne bacteria. In addition, the climate control unit within the general ventilation system of barn 1 was defective for four weeks (week 2 until week 4 as indicated in Table 4). There was not any noticeable effect during that time but the overall mean indoor temperature was slightly higher compared to barn 2 which might have had an influence on dust formation and airborne bacterial load.

With the UVC module in operation in-room, total dust concentrations in barn 1 were reduced by 13.5% to 22% which is less than others reported [10,52,54]. However, others used different air flow rates through the filter. It is known that, besides other factors, the air exchange rates strongly influence dust concentrations [12]. The ventilation rate in both barns was 1,200 m^3^/h (air exchange rate of approximately 17-fold/h) and the UVC module in barn 1 accounted for additional six times air exchange. The air flow patterns of supply air were visualized using artificial fog and revealed a laminar air flow which was not disturbed by the UVC module and vice versa. Nevertheless, the three sampling locations chosen might have been suboptimal with regard to spatial variability of airborne dust in animal housings [55]. We did not investigate on the latter aspect which can be seen as a weakness of our study. Moreover, dust deposition rates were reported to be quite high and largely contribute to dust clearance especially in housing with fully slatted floors [52]. This is probably the reason why recirculating air filtration only resulted in a remote reduction of dust compared to airborne bacteria [52]. In contrast to the results obtained by air samplings taken at height of the animals, measures taken in close proximity to the UVC module revealed an up to 99.5% reduction of bacteria. This is similar to findings of Schulz and coworkers who examined UV-irradiation combined to an air washer [12].

In both trials, lung health assessed after slaughter revealed no distinct differences between pigs of barn 1 (with UVC module) and pigs from barn 2 (reference barn). Although, previous investigations showed a positive impact on animal lung health in pig housings with recirculating air filtration [9,11,14], these findings could not be substantiated by our research. The main reason, and concurrently a major weakness of our study, might be the small number of animals used in our experimental setup which restricted a statistical evaluation on this subject. Concentrations of CO_2_ and NH_3_ were always below the threshold limit values recommended by German order [33].

The amount and nature of airborne dust and microorganisms affects pig and human health as well as animal performance [9,11,49]. Moreover, the emission of infectious microbes via bioaerosols is of significant concern for public and environmental health as high numbers of e.g. methicillin-resistant *S*. *aureus* have been found in air sampling from pig facilities [14,56]. Hence, the reduction of airborne dust and microorganisms in animal confinements will decrease the risk of harmful effects on animals and humans [9,50,57]. By doing so, the emission ratio of these pollutants will also decrease thereby enhancing environmental protection. Based on the German Federal Immission Control Act (BImSchG) legal guidelines for exhaust air purification have been established in some German Federal States depending on the type of livestock and number of animal places involved but exhaust air treatment is not commonly used in all pig confinements. Our results demonstrate, that indoor air filtration, especially when combined to UVC irradiation, can significantly reduce airborne bacteria and lowers dust burden indoors which further contributes to environmental protection.

## Conclusion

We successfully demonstrated the effectiveness of air disinfection using UVC irradiation at laboratory scale with reduction efficiencies of >99% for certain viruses and bacteria. Moreover, combining UVC irradiation to recirculating air filtration proved to be successful in reducing airborne bacteria and dust in a small animal facility. The implementation of such devices might improve the overall environmental quality in animal facilities.

## Supporting information

S1 File UVGI simulation within the filter test chamber at a relative humidity of 20%. The upper left graph depicts a scheme of the filter test chamber and the positions of the two UVC tubes are given as blue lines. The colored graphs display the UVC intensity (μW/cm^2^) within the filter test chamber.
(PDF)

S2 File UVGI simulation within the filter test chamber at a relative humidity of 66%. The upper left graph depicts a scheme of the filter test chamber and the positions of the two UVC tubes are given as blue lines. The colored graphs display the UVC intensity (μW/cm^2^) within the filter test chamber.
(PDF)
