# Supplementary material for: Impact of UVC-sustained recirculating air filtration on airborne bacteria and dust in a pig facility
Source: PLoS One. 2019 Nov 7;14(11):e0225047. doi: 10.1371/journal.pone.0225047 (PMC6837447; doi:10.1371/journal.pone.0225047)
Supplement: S1 File — The upper left graph depicts a scheme of the filter test chamber and the positions of the two UVC tubes are given as blue lines. The colored graphs display the UVC intensity (μW/cm2) within the filter test chamber. (PDF) [file pone.0225047.s001.pdf]

UVGI CALCULATION

Project Information

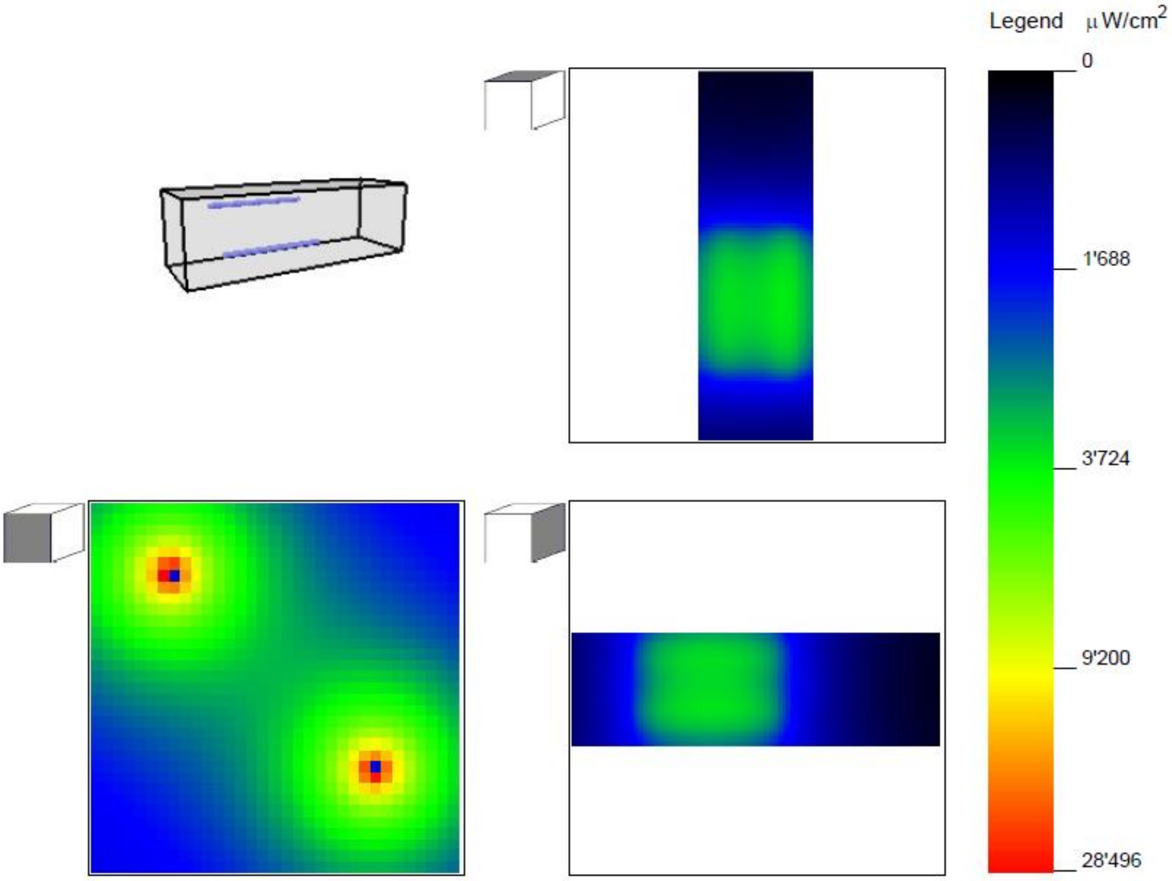

Minimal Intensity:	45	µW/cm ^{2}
Maximal Intensity:	28'396	µW/cm ^{2}
Average Intensity:	1'612	µW/cm ^{2}
Air Volume:	1800.0	m ^{3}/h
Time of Exposure:	1.13	s
Air Temperature:	20.0	°C
Relative Humidity [RH]:	20	%
